# Solitary facial lesion of orf: An unusual presentation

**DOI:** 10.14744/nci.2020.59254

**Published:** 2021-11-18

**Authors:** Muge Gore Karaali, Ayse Esra Koku Aksu, Asude Kara Polat, Mehmet Salih Gurel

**Affiliations:** 1. Department of Dermatology, Erzincan Binali Yildirim University, Mengucek Gazi Training and Research Hospital, Istanbul, Turkey; 2. Department of Dermatology, University of Health Science, Istanbul Training and Research Hospital, Istanbul, Turkey; 3. Department of Dermatology, Medeniyet University Faculty of Medicine, Goztepe Training and Research Hospital, Istanbul, Turkey

**Keywords:** Face, orf, parapoxvirus

## Abstract

Orf, ecthyma contagiosum, is a zoonotic viral infectious disease caused by parapoxvirus that affects particularly sheep and goats. Human may be infected with direct contact with contagious animals or by handling contaminated animal products. Lesions are localized mostly on the hands and fingers, but atypical localizations such as head or face have been rarely reported. Herein, we report a case of orf disease on the eyebrow with clinical follow-up images. Physicians should be aware of the possibility of this entity based on contact anamnesis with infected animals and clinical appearance.

**O**rf is a zoonotic viral infectious disease, caused by parapoxvirus that affects particularly sheep and goats. Human may be infected when their eroded skin have direct contact with contagious animal lesions [1]. It usually presents as solitary, erythematous papule which progress through six clinical stages [2]. The most frequent localization of the lesions is hands and fingers, but atypical localizations had been reported in the literature [1, 3–7].

Orf disease is diagnosed with clinical history and lesion characteristics, and evolutionary progress of the disease. Histopathological examination, viral culture, detection of virus antigen and antibodies, polymerase chain reaction, electron microscopic examination, and immunofluorescence examination may be used as diagnostic techniques when needed [7]. The orf lesion usually recovers spontaneously without any specific treatment. Beyond non-specific wound care, antibiotics for secondary infection, topical imiquimode, idoxuridine, and systemic interferon alfa injections may be used if necessary [2, 3, 8].

Herein, we report a case of orf disease on the eyebrow with clinical follow-up images.

## Case Report

**Figure 1.**
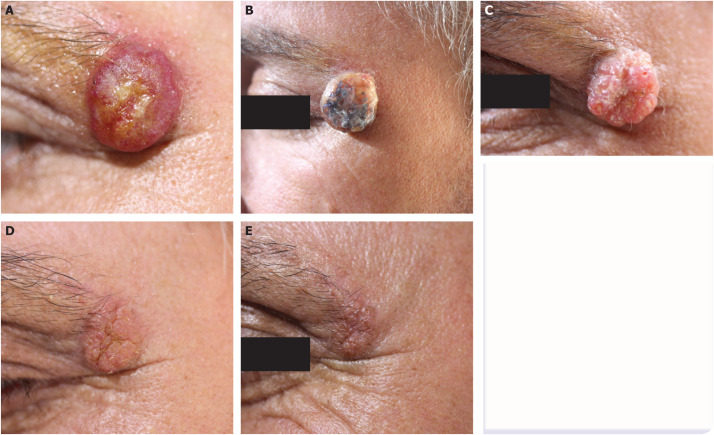
**(A)** Lesion on acute stage. A 3 cm well-defined, erythematous, edematous, centrally ulcerated, hemorrhagic moderately hard, and fragile nodule at the left eyebrow tip, **(B)** Lesion on regenerative stage. A 3.5 cm hemorrhagic crusted nodule after cauterization, **(C)** Lesion on papillomatous stage. A 3 cm erythematous and fragile nodule with papillomatous projections, **(D)** Lesion on regression stage. A 2 cm skin-colored plaque with mild and superficially papillomatous projections, **(E)** Healed lesion.

A 54-year-old otherwise healthy man presented with a bleeding mass at his left eyebrow tip for 2 weeks in the dermatology outpatient clinic. He had a history of suturing after traumatic erosion by a sheep 1 week before the appearance of the lesion at the feast of sacrifice. Dermatologic examination revealed a 3 cm well-defined, erythematous, edematous, centrally ulcerated, hemorrhagic, moderately hard, and fragile nodule at the left eyebrow tip (Fig. 1A).

Dermatologic examination and clinical history of a trauma by a sheep led us the clinical presumptive diagnosis of orf disease and therefore the patient decided to be followed up without intervention. Due to the possibility of bacterial superinfection, topical fusidic acid ointment was recommended.

However, the patient applied to another dermatology outpatient clinic due to persisted and even increasing bleeding mass. He was diagnosed with pyogenic granuloma, and therefore treated with cauterization superficially. Due to the persisted lesion, he reapplied to our dermatology outpatient clinic and he was advised to avoid any surgical intervention because of the aggravation. He was followed up with wet dressing and fusidic acid ointment. The photographs of patients were recorded at the follow-up visits (Fig. 1B–E). The patient was continued to be followed up without any intervention because of regression of the lesion at the follow-up visits. The lesion resolved spontaneously in 6 weeks without any scarring.

## Discussion

Orf disease, known as ecthyma contagiosum, is a viral zoonotic infectious disease caused by parapoxvirus which actually leads periorificial contagious lesions in the skin of sheep and goats [3]. Direct contact with contagious animal lesions can cause the disease if damaged skin integrity is present after an incubation period of 1 week [1, 2]. In Turkey like other Muslim communities, many cases are seen after the feast of sacrifice [3]. In our case, the patient applied to our outpatient clinic following the feast of sacrifice and his lesion was appeared approximately 1 week after the erosive trauma by a sheep in concordance with incubation period of the virus.

The lesions in human are located mostly on hands and fingers due to the most contact places with infected lesions [3]. Face localization has been reported occasionally in the literature [1, 3–5, 7]. Our case’s lesion was located on the face at the left eyebrow tip like a paper reporting a case over the right eyebrow following a traumatic erosion and contact with a lamb while fading [1].

For definitive diagnosis auxiliary techniques, such as histopathology and viral examination may be used, however clinical history, lesion characteristics, and evolutionary progress of the disease are sufficient for making the diagnosis. Histopathological examination is not necessary but if performed epidermal necrosis, vacuolization in keratinocytes, mixed dermal infiltration, finger like projections of the epidermis into the dermis, and eosinophilic inclusion bodies may be seen according to the clinical stage of the lesion [2].

The lesion evolves through six clinical stages: Maculopapular stage, targetoid stage, acute stage (weeping nodule), regenerative dry stage with black dots, papillomatous stage, and regression stage with a dry crust [2]. In our case, disease was on the acute stage during application to our clinic (Fig. 1A). Papillomatous and regression stages were observed and showed in Figure 1C and d; but regenerative stage was not typical with black dots because of cauterization of the lesion (Fig. 1B).

Awareness of this disease is important especially on endemic regions. An unnecessary intervention such as curettage, cauterization, laser, and surgical excision may cause exacerbation of the disease as reported in the literature [7, 8]. Pyogenic granuloma, cutaneous metastases, anthrax, Milker’s nodule, keratoacanthoma, and giant molluscum may be thought for differential diagnoses [9]. Following the feast of sacrifice, in concordance with incubation period of 1 week and typical clinical appearance and evolution of the lesion, with no accompanying disease of the patient led us to rule out the differentials. Our patient was misdiagnosed in another dermatology outpatient clinic with pyogenic granuloma because of clinical similarity of the lesion and intervened with cauterization due to bleeding. Except for mild erythema and edema, no exacerbation was observed. Although the absence of histopathological confirmation is the lack of this case, because of the presumptive diagnosis of orf, we do not want to perform any interventions because of the possibility of exacerbation of the disease. The contact history of the patient and the observation of the typical orf course in the follow-up of the lesion prevented us from performing additional examinations. Regression of the lesion in accordance with the orf course in clinical follow-up also supported us.

Orf disease heals generally without any specific treatment with no or mild scarring, but for immunocompromised patients specific therapies can be used [2, 3, 8]. The lesion in our case regressed in 6 weeks without any specific treatment except for wound care and fusidic acid ointment for possible secondary bacterial infection.

### Conclusion

Orf disease is a zoonotic viral infectious disease with a predilection for hands and fingers. Because of infrequency of this disease on the face, we report a case of orf disease on eyebrow with clinical follow-up images. Physicians should be aware of the possibility of this entity based on contact anamnesis with infected animals and clinical appearance even in unexpected localizations.
